# One Health Spread of 16S Ribosomal RNA Methyltransferase-Harboring Gram-Negative Bacterial Genomes: An Overview of the Americas

**DOI:** 10.3390/pathogens12091164

**Published:** 2023-09-15

**Authors:** Fábio Parra Sellera, Danny Fuentes-Castillo, João Pedro Rueda Furlan

**Affiliations:** 1 Department of Internal Medicine, School of Veterinary Medicine and Animal Science, University of São Paulo, São Paulo 05508-270, Brazil; fsellera@usp.br; 2 School of Veterinary Medicine, Metropolitan University of Santos, Santos 11065-402, Brazil; 3 Departamento de Patología y Medicina Preventiva, Facultad de Ciencias Veterinarias, Universidad de Concepción, Chillán 3780000, Chile; dannyfuentes@udec.cl; 4 Department of Clinical Analyses, Toxicology and Food Science, School of Pharmaceutical Sciences of Ribeirão Preto, University of São Paulo, Ribeirão Preto 14040-903, Brazil

**Keywords:** 16S-RMTase, *Acinetobacter baumannii*, aminoglycoside resistance, *armA*, *Enterobacterales*, One Health, *Pseudomonas aeruginosa*, *rmtB*, *rmtF*

## Abstract

Aminoglycoside antimicrobials remain valuable therapeutic options, but their effectiveness has been threatened by the production of bacterial 16S ribosomal RNA methyltransferases (16S-RMTases). In this study, we evaluated the genomic epidemiology of 16S-RMTase genes among Gram-negative bacteria circulating in the American continent. A total of 4877 16S-RMTase sequences were identified mainly in *Enterobacterales* and nonfermenting Gram-negative bacilli isolated from humans, animals, foods, and the environment during 1931–2023. Most of the sequences identified were found in the United States, Brazil, Canada, and Mexico, and the prevalence of 16S-RMTase genes have increased in the last five years (2018–2022). The three species most frequently carrying 16S-RMTase genes were *Acinetobacter baummannii*, *Klebsiella pneumoniae*, and *Escherichia coli*. The *armA* gene was the most prevalent, but other 16S-RMTase genes (e.g., *rmtB*, *rmtE*, and *rmtF*) could be emerging backstage. More than 90% of 16S-RMTase sequences in the Americas were found in North American countries, and although the 16S-RMTase genes were less prevalent in Central and South American countries, these findings may be underestimations due to limited genomic data. Therefore, whole-genome sequence-based studies focusing on aminoglycoside resistance using a One Health approach in low- and middle-income countries should be encouraged.

## 1. Introduction

The burden of antimicrobial resistance (AMR) has generated tremendous obstacles for global public health authorities [1]. Due to their ability to acquire multiple resistance mechanisms, antimicrobial-resistant bacteria can jeopardize the effectiveness of commercially available antimicrobials [2]. Indeed, multidrug-resistant (MDR) bacteria have emerged and rapidly spread worldwide; they are expected to be one of the top-ranked causes of death in the future [1]. Furthermore, AMR has been no longer limited to human settings and there are increasing reports of medically relevant bacteria in a broad range of non-human hosts and anthropogenically impacted environments, favoring its spread and inter-host transmission in a continuous cycle. In this context, the One Health approach, which recognizes the interconnectedness of human, animal, and environmental health, has gained international recognition as an essential strategy to tackle the AMR crisis since this red-alert global health threat is now circulating among different sectors [3].

Aminoglycosides are broad-spectrum antimicrobials that trigger the inhibition of protein synthesis in bacteria [4,5]. This antimicrobial class was first introduced in clinical practice in the 1940s, but its use was rapidly reduced and replaced by other antimicrobials, including β-lactams and fluoroquinolones [6,7]. Conversely, with the increasing rates of resistance to these antimicrobials, aminoglycosides returned to the spotlight as a possible synergistic therapeutic option for Gram-negative bacterial infections [5,7]. Worryingly, aminoglycoside resistance has emerged via two main mechanisms, including the production of aminoglycoside-modifying enzymes (AMEs) and 16S ribosomal RNA methyltransferases (16S-RMTases). Although there are different 16S-RMTase-encoding genes, *armA* and *rmtB* have been most frequently reported, being identified in different medically important Gram-negative bacteria worldwide [7,8].

In this study, we performed a genomic epidemiology investigation to address the One Health dissemination of 16S-RMTase-producing Gram-negative bacteria in the Americas. We analyzed the most prevalent 16S-RMTase genes and identified the most common bacterial species carrying these genes. Additionally, we examined the distribution of 16S-RMTase-positive bacteria in all countries of the Americas and highlighted the main origins and isolation sources of these bacteria. We also evaluated the temporal trends of the spread of 16S-RMTase genes over the last few decades.

## 2. Materials and Methods

### Data Collection

To determine the distribution of 16S-RMTase-producing Gram-negative bacteria recovered from different sources of origin and countries of the Americas (North, Central, and South), bacterial genomes harboring the most important 16S-RMTase genes were included. The search strategy was performed using the NCBI National Database of Antibiotic Resistant Organisms [9] from inception until 23 June 2023. The genes belonging to the 16S rRNA (adenine(1408)-N(1))-methyltransferase (*npmA* and *npmB*) and 16S rRNA (guanine(1405)-N(7))-methyltransferase (*armA*, *rmtA*, *rmtB*, *rmtC*, *rmtD*, *rmtE*, *rmtF*, *rmtG*, and *rmtH*) families were searched individually. The records were downloaded and turned into a Microsoft Office Excel^®^ spreadsheet file. A detailed screening was carried out by first filtering the field “Location”. Subsequently, the fields “Bacterial species”, “Year”, and “Host (referred to as origin in this study) and/or Isolation source” were analyzed. The bacterial genomes were grouped according to origin (isolation source) as follows: humans; animals (companion, food-producing, and wildlife); food; and the environment (water and sediment). Afterwards, duplicate strains and sequences with <97% coverage and <99% identity of reference were removed. Finally, genomic data were interpreted and discussed.

## 3. Results

### 3.1. Identification of 16S-RMTases

**Figure 1:**
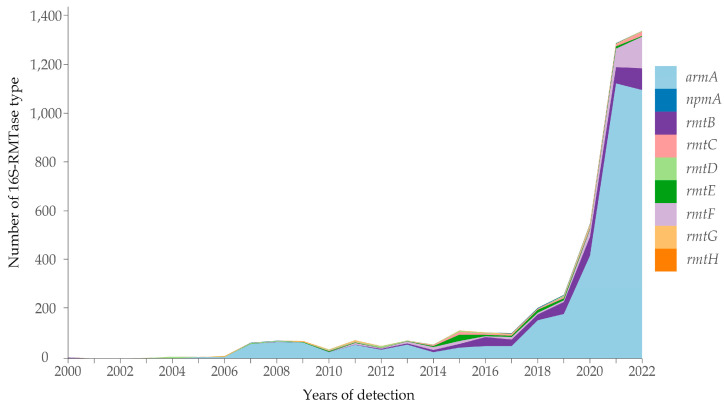
Temporal distribution of 16S-RMTase genes during 2000–2022 in the Americas.

All 16S-RMTase genes searched were identified, except *rmtA* and *npmB*. These genes totaled 4877 sequences from bacterial genomes of *Enterobacterales*, nonfermenting Gram-negative bacilli, and *Clostridioides difficile* obtained from humans, animals (companion animals (horses and dogs), food-producing animals (cattle, pigs, and turkeys), and wildlife (gulls), food (product-raw-ground, comminuted, and otherwise nonintact from chicken, pig, and turkey, and papaya), and the environment (river, sink drain/drain, sediment, sewage, and wastewater) during 1931–2023. In the last five years (2018–2022), 3662 bacterial genomes were identified as carrying 16S-RMTase genes, representing four times more than that found in genomes from 2000 to 2017 (Figure 1). In 2023 (until June), 290 16S-RMTase-positive genomes have been detected. Curiously, two genomes of *armA*-producing *Acinetobacter baumannii* (BioSample: SAMN18636604) and *rmtD1*-positive *Pseudomonas aeruginosa* (BioSample: SAMN04455104) from 1931 and 1997, respectively, were also identified, constituting the oldest genomes containing 16S-RMTase genes (data not presented in Figure 1).

We identified that 16S-RMTase genes have been carried by a broad range of Gram-negative bacterial species, highlighting those of clinical interest, such as *Escherichia coli*, *Klebsiella pneumoniae* complex, *Enterobacter cloacae* complex, *Salmonella enterica*, *P. aeruginosa*, and *A. baumannii*. On the other hand, these genes were also identified in environmental or unusual species, including *Aeromonas hydrophila* and *Pluralibacter gergoviae*. *K. pneumoniae* was the most prevalent species carrying *rmtB*, *rmtC*, *rmtF*, *rmtG*, and *rmtH* genes, while *A. baumannii*, *C. difficile*, *P. aeruginosa*, and *S. enterica* were the most common species harboring *armA*, *npmA*, *rmtD*, and *rmtE* genes, respectively. Overall, the three most frequent species carrying 16S-RMTase genes were *A. baummannii*, *K. pneumoniae*, and *E. coli*.

**Figure 2:**
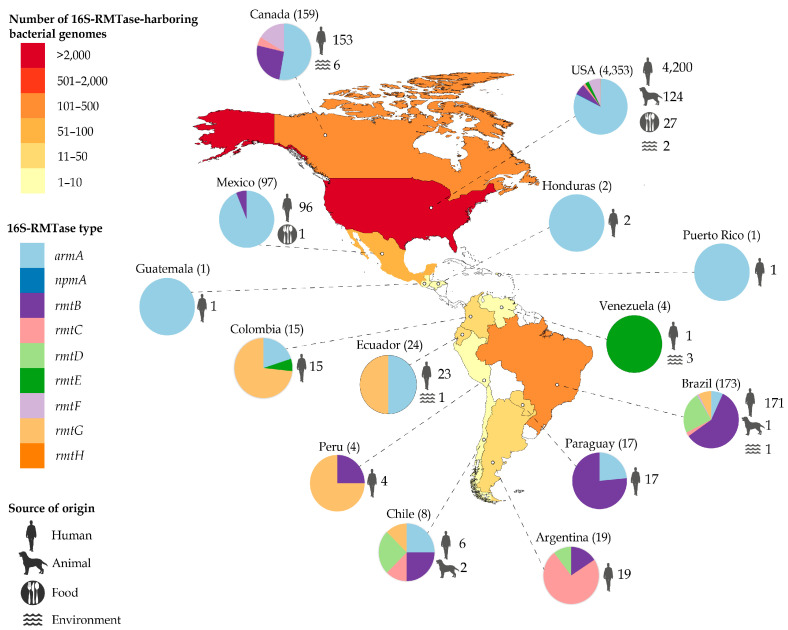
Geographical distribution of 16S-RMTase-harboring bacterial genomes from 1931 to June 2023 in the Americas. Colored countries show quantity of 16S-RMTase-harboring bacterial genomes found. Circle charts show the presence of the 16S-RMTase types present in each country. Sources of origins were grouped into human, animal, food, or environmental samples.

The 16S-RMTase genes were predominantly in countries of North America (*n* = 4353, 94.5%), followed by South America (5.4%) and Central America (0.1%). The United States (USA) contained most of the sequences obtained (*n* = 4353, 89.2%), followed by Brazil (*n* = 173, 3.5%), Canada (*n* = 159, 3.3%), and Mexico (*n* = 97, 2.0%). Regarding the origins, humans were predominant (*n* = 4709, 96.5%), followed by animals (*n* = 127, 2.6%), foods (*n* = 28, 0.6%), and the environment (*n* = 13, 0.3%) (Figure 2). Among animal-associated 16S-RMTase genes, higher prevalence was in pigs, followed by horses, cattle, dogs, turkeys, and gulls.

### 3.2. Prevalence of 16S-RMTases

The *armA* gene was the most prevalent with 3797 (77.9%) sequences identified in *A. baumannii*, *Citrobacter amalonaticus*, *Citrobacter koseri*, *Enterobacter hormaechei*, *E. coli*, *Klebsiella aerogenes*, *Klebsiella oxytoca*, *K. pneumoniae*, *Klebsiella quasipneumoniae*, *Morganella morganii*, *P. gergoviae*, *Proteus mirabilis*, *Providencia rettgeri*, *Raoultella ornithinolytica*, and *S. enterica* from Brazil, Canada, Chile, Colombia, Ecuador, Guatemala, Honduras, Mexico, Paraguay, Puerto Rico, and the USA.

For the *rmtB* gene, 445 (9.1%) sequences, including *rmtB1*, *rmtB2*, and *rmtB4*, were detected in *E. coli*, *Citrobacter freundii*, *Citrobacter werkmanii*, *E. hormaechei*, *K. pneumoniae*, *M. morganii*, *P. rettgeri*, *P. aeruginosa*, and *S. enterica* from Argentina, Brazil, Canada, Chile, Mexico, Paraguay, Peru, and the USA. The *rmtF* gene with 347 (7.1%) sequences was detected in *K. pneumoniae*, *P. aeruginosa*, and *S. enterica* from Brazil, Canada, and the USA. For the *rmtE* gene, 96 (2.0%) sequences were identified in *A. baumannii*, *E. coli*, *K. pneumoniae*, *K. quasipneumoniae*, and *S. enterica* from Colombia, the USA, and Venezuela. Interestingly, *armA*, *rmtB*, *rmtE*, and *rmtF* genes were the only ones detected in all target origins.

Ninety-three (1.9%) *rmtC* sequences were identified in *C. freundii*, *C. koseri*, *E. coli*, *Enterobacter cloacae*, *E. hormaechei*, *K. pneumoniae*, *P. mirabilis*, *P. rettgeri*, and *S. enterica* from Argentina, Brazil, Canada, Chile, and the USA. For the *rmtD* gene, 46 sequences (0.9%), including *rmtD1* and *rmtD2* sequences, were found in *A. hydrophila*, *C. koseri*, *E. hormaechei*, *E. coli*, *K. pneumoniae*, *K. quasipneumoniae*, and *P. aeruginosa* from Argentina, Brazil, Chile, and the USA. Forty *rmtG* gene sequences (0.8%) were found in *K. aerogenes*, *K. pneumoniae*, *K. quasipneumoniae*, *K. variicola*, *E. cloacae*, *E. hormaechei*, *E. kobei*, *E. coli*, *P. stuartii*, and *P. aeruginosa* from Brazil, Chile, Colombia, Ecuador, and Peru. Finally, *npmA* (*n* = 11, 0.2%) and *rmtH* (*n* = 3, 0.06%) sequences were detected in *C. difficile* and *K. pneumoniae* from the USA. The *npmA*, *rmtH*, *rmtC* genes were only identified in humans. The *rmtD* gene was found in humans, animals, and the environment, while the *rmtG* gene was detected in humans and the environment.

## 4. Discussion

In this study, to the best of our knowledge, we present the first description and distribution analysis of 16S-RMTase-producing Gram-negative bacterial genomes in the Americas circulating among humans, animals, foods, and the environment. These data allow a comprehensive landscape of the spread of 16S-RMTase-producing bacteria in all countries of North, South, and Central America. Further comparative analyses with other continents would be valuable for a better understanding of the global prevalence, genomic features, and main drivers of 16S-RMTase genes.

Remarkably, our findings shed light on the rise in the *armA* gene harbored by Gram-negative bacteria of clinical interest, including *A. baumannii* and several species of *Enterobacterales*. Although the other 16S-RMTase genes were less prevalent, these genes could have been underestimated due to limited genomic data on Central and South America. Indeed, more than 90% of 16S-RMTase sequences in the Americas were found in North America, which could be explained by higher investments in the genomic epidemiology surveillance of antimicrobial-resistant bacteria, especially in the USA and Canada.

Another important issue is the One Health dissemination of these bacteria. Although our findings revealed that 16S-RMTase-positive bacteria are primarily related to humans, where studies are concentrated, we identified that this problem is not limited to human settings. In this regard, several sequences obtained from bacteria isolated from companion and food-producing animals were identified. In fact, aminoglycosides have been used for oral, parenteral, and topical applications in animals, being the sixth most commonly used antimicrobial class in veterinary medicine [10], which might contribute to the selective pressure of aminoglycoside resistance genes.

We also highlight the presence of 16S-RMTase-producing bacteria in food products, which could pose the risk of humans and animals acquiring these medically important organisms through the consumption of contaminated food. More critically, the occurrence of these bacteria in wildlife and the environment might suggest that these pathogens are transgressing hospital settings and then spilling over into nature, possibly exacerbated by anthropogenic pollution (e.g., rural sewage and hospital wastewater) [11].

Considering aminoglycosides remains valuable therapeutic options for human and veterinary medicine, our findings highlight the urgent need for a One Health view of the emergence of 16S-RMTase-positive bacteria. Indeed, the globalization and interconnectedness of different sectors that embrace the human–animal–environment interface require strengthening the surveillance of aminoglycoside resistance, mitigation strategies, and One Biosecurity policies [12].

Gram-negative bacteria stand out as the most relevant pathogens that can harbor broad armamentariums against medically important antimicrobials, including aminoglycosides, β-lactams, and fluoroquinolones [13]. Currently, infections caused by MDR Gram-negative bacterial pathogens are challenging and represent a critical issue in the field of infectious diseases due to the limited therapeutic options available. In this regard, aminoglycosides have been positioned as priority antimicrobials for human medicine, but the emergence and spread of MDR and aminoglycoside-resistant bacteria, including *K. pneumoniae*, *E. coli*, *A. baumannii*, and *P. aeruginosa*, have raised concerns [14].

Among the two main acquired mechanisms of aminoglycoside resistance, the 16S-RMTases are more clinically significant than AMEs since they ensure high-level resistance to clinically relevant aminoglycosides belonging to the 4,6-disubstituted 2-deoxystreptamine (DOS) group [15,16]. The different 16S-RMTases catalyze specific nucleotides in the 16S rRNA component of the bacterial ribosome. Specifically, the 16S-RMTases add a methyl group to specific adenine or cytosine residues in the 16S rRNA, affecting the binding sites for aminoglycosides, and thus preventing the antimicrobials from effectively interacting with the ribosome [17].

Interestingly, a novel aminoglycoside agent, named plazomicin, is not effective against strains that produce acquired 16S-RMTases. To date, most *Klebsiella pneumoniae* carbapenemase (KPC)-producing *K. pneumoniae* strains remain susceptible to plazomicin, but the emergence of KPC and 16S-RMTase co-producing strains could be a problem on the horizon [18]. Worryingly, the occurrence of plazomicin-resistant *Enterobacterales* co-producing carbapenemases and 16S-RMTases has been documented [19]. Therefore, the presence of 16S-RMTases in Gram-negative bacterial pathogens should be considered an important issue that significantly limits the effectiveness of aminoglycosides in treating infections caused by these resistant bacteria [18].

The coexistence of 16S-RMTase genes and β-lactamase-encoding genes (e.g., extended-spectrum β-lactamases and carbapenemases) or *mcr* genes have been reported around the world [20,21,22]. In Brazil, the coexistence of *rmtD* and *bla*_SPM-1_ was identified in a clinical *P. aeruginosa* strain [23], while in Bolivia, clinical *K. pneumoniae* strains co-harboring *rmtB* and *bla*_CTX-M-65_ were described [24]. In addition, the co-occurrence of 16S-RMTases and New Delhi metallo-β-lactamases in bacterial pathogens has been considered an important AMR phenomenon and needs to be paid closer attention by scientific and global health authorities [21,25]. In this study, the strategy used did not allow for the evaluation of the coexistence of 16S-RMTases and other clinically important AMR mechanisms, limiting this discussion.

Although the 16S-RMTase genes are mainly mediated by transposons and, consequently, embedded into plasmids or chromosomes [7,8], the complete characterization of their genetic environments does not seem to be a priority in studies in the Americas since most studies are PCR-based. In comparison with European and Asian countries, Latin American countries continue to have a lower availability of whole-genome sequences of bacterial strains, limiting robust genomic analyses. In addition, as the most available genomes were assembled using short-read sequencing, it was not possible to determine the exact location of 16S-RMTase genes, which was another limitation of this study. Therefore, a hybrid sequencing strategy should be encouraged for a better understanding of acquired aminoglycoside resistance.

As an initial screening step for 16S-RMTases, specific resistance phenotypes should be looked at closely. NpmA- or NpmB-positive strains have resistance to monosubstituted DOS (apramycin), 4,5-disubstituted DOS (neomycin), and 4,6-disubstituted DOS (gentamicin, tobramycin, and amikacin), but are susceptible to agents with no DOS ring (streptomycin), being classified as having pan-aminoglycoside resistance. Strains carrying ArmA and RmtA-H present resistance to 4,6-disubstituted DOS but susceptibility to monosubstituted DOS, 4,5-disubstituted DOS, and no DOS ring [18,26]. Therefore, studies focusing on aminoglycoside resistance screening with subsequent whole-genome-sequence-based analysis in low- and middle-income countries should be encouraged.

## 5. Conclusions

In summary, we present genomic epidemiological data of 16S-RMTase genes in the Americas. The *armA* gene was the most prevalent gene, but the other 16S-RMTase genes could be silently emerging. A broad range of Gram-negative bacteria have been encoding these genes, but *A. baumannii*, *K. pneumoniae*, *E. coli*, and *P. aeruginosa* deserve special attention since these bacteria are in the spotlight as important nosocomial pathogens. The higher prevalence of 16S-RMTase genes in North America might be associated with the strengthening genomic surveillance of AMR in high-income countries. Finally, since our data revealed that 16S-RMTase genes are spreading in different sectors in American countries, a One Health view should be applied to tackle the aminoglycoside resistance problem.

## Data Availability

Data Availability Statements are available in section “MDPI Research Data Policies” at https://www.mdpi.com/ethics (accessed on 25 July 2023).
